# Identification and characterization of Nanobodies targeting the EphA4 receptor

**DOI:** 10.1074/jbc.M116.774141

**Published:** 2017-05-19

**Authors:** Lies Schoonaert, Laura Rué, Bart Roucourt, Mieke Timmers, Susan Little, Lucía Chávez-Gutiérrez, Maarten Dewilde, Peter Joyce, Adam Curnock, Peter Weber, Jurgen Haustraete, Gholamreza Hassanzadeh-Ghassabeh, Bart De Strooper, Ludo Van Den Bosch, Philip Van Damme, Robin Lemmens, Wim Robberecht

**Affiliations:** From the ‡ KU Leuven-University of Leuven, Department of Neurosciences, Experimental Neurology and Leuven Research Institute for Neuroscience and Disease (LIND), 3000 Leuven, Belgium,; § VIB, Center for Brain and Disease Research, Laboratory of Neurobiology, 3000 Leuven, Belgium,; ¶ KU Leuven-University of Leuven, Laboratory for Signal Integration in Cell Fate Decision, 3000 Leuven, Belgium,; ‖ Vertex Pharmaceuticals (Europe) Ltd., Biology Department, OX14 4RW Abingdon, United Kingdom,; ** KU Leuven, Department of Neurosciences and Leuven Research Institute for Neuroscience and Disease (LIND), 3000 Leuven, Belgium,; ‡‡ Protein Service Facility, Inflammation Research Center, VIB, Ghent University, 9052 Ghent, Belgium,; §§ Nanobody Service Facility, VIB, Vrije Universiteit Brussel, 1050 Brussels, Belgium,; ¶¶ Institute of Neurology, University College London, WC1E 6BT London, United Kingdom, and; ‖‖ University Hospitals Leuven, Department of Neurology, 3000 Leuven, Belgium

**Keywords:** phosphorylation, protein-protein interaction, receptor-tyrosine kinase, regeneration, single-domain antibody (sdAb,Nanobody), EphA4, VHH, inhibition, ligand-binding domain

## Abstract

The ephrin receptor A4 (EphA4) is one of the receptors in the ephrin system that plays a pivotal role in a variety of cell-cell interactions, mostly studied during development. In addition, EphA4 has been found to play a role in cancer biology as well as in the pathogenesis of several neurological disorders such as stroke, spinal cord injury, multiple sclerosis, amyotrophic lateral sclerosis (ALS), and Alzheimer's disease. Pharmacological blocking of EphA4 has been suggested to be a therapeutic strategy for these disorders. Therefore, the aim of our study was to generate potent and selective Nanobodies against the ligand-binding domain of the human EphA4 receptor. We identified two Nanobodies, Nb 39 and Nb 53, that bind EphA4 with affinities in the nanomolar range. These Nanobodies were most selective for EphA4, with residual binding to EphA7 only. Using Alphascreen technology, we found that both Nanobodies displaced all known EphA4-binding ephrins from the receptor. Furthermore, Nb 39 and Nb 53 inhibited ephrin-induced phosphorylation of the EphA4 protein in a cell-based assay. Finally, in a cortical neuron primary culture, both Nanobodies were able to inhibit endogenous EphA4-mediated growth-cone collapse induced by ephrin-B3. Our results demonstrate the potential of Nanobodies to target the ligand-binding domain of EphA4. These Nanobodies may deserve further evaluation as potential therapeutics in disorders in which EphA4-mediated signaling plays a role.

## Introduction

Ephrin receptors, the largest family of receptor-tyrosine kinases, are subdivided in A- and B-class receptors. In mammals there are nine EphA receptors (EphA1-EphA8, EphA10) that interact with five ephrin-A ligands (ephrin-A1–ephrin-A5) and five EphB receptors (EphB1–EphB4, EphB6) that interact with three ephrin-B ligands (ephrin-B1–ephrin-B3) (1). There is some interclass promiscuity, as EphA4 can also interact with ephrin-B ligands, whereas EphB2 also interacts with ephrin-A5 (2–4).

The ephrin system plays a major role in a variety of cell-cell interactions. In the developing nervous system it is pivotal as an axonal guidance system, whereas in the adult brain it is involved in synaptic plasticity and long-term potentiation (5, 6). Importantly, the ephrin system also plays a role in cancer biology and in the pathogenesis of several neurological disorders (5). EphA4 is up-regulated in spinal cord injury, traumatic brain injury, and stroke, and blocking of the receptor increases functional recovery in models for these conditions (7–9). Interestingly, antagonism of EphA4 improves long-term potentiation defects in a mouse model for Alzheimer's disease and improves outcome in animal models for amyotrophic lateral sclerosis (ALS)^9^ and stroke (9–12). EphA4 expression is inversely correlated with survival time in ALS patients, which suggests that EphA4 is also involved in ALS human pathology (11). These findings suggest that EphA4 inhibition may have the potential for the treatment of neurological disorders.

Inhibition of EphA4 signaling can be accomplished by targeting the ATP-binding pocket in the kinase domain or by blocking the interaction with ephrin ligands (13, 14). Because the ATP-binding pocket is highly conserved among tyrosine kinases it is very difficult to develop selective inhibitors (13). Alternatively targeting the ligand-binding domain (LBD) certainly allows the development of more selective compounds, but this poses several other problems. On one hand, the protein interaction surface to be covered is large; 900 Å^2^ in the case of EphA4 (15, 16). On the other hand, the LBD is dynamic by nature, as EphA4 can adopt a conformation similar to other EphA receptors upon interaction with ephrin-A ligands or characteristics of EphB receptors when interacting with ephrin-B ligands (2, 4, 17).

Nevertheless several peptides and small molecules have been identified that bind the EphA4 LBD and block its interaction with ephrin ligands (18–22). One of these EphA4 antagonists is the KYL peptide, which has been extensively characterized and shown to be effective in several *in vitro* assays as well as in *in vivo* spinal cord injury and ALS models (11, 19, 23, 24), suggesting the potential of an EphA4-based therapeutic approach.

The aim of this study was to develop highly selective and potent EphA4 inhibitors. To achieve this goal, we took advantage of the Nanobody technology (25–27). Nanobodies (Nbs) or VHHs are small antigen-binding fragments derived from camelid heavy-chain-only antibodies that are devoid of light chains. They are superior to conventional antibodies in terms of stability, solubility, and immunogenicity (27). Furthermore, they are much smaller than conventional antibodies (12–15 kDa *versus* 150–160 kDa) and can penetrate small clefts and cavities (28).

We were able to generate Nbs against the LBD of the EphA4 receptor. Two of these Nbs specifically bind the EphA4 receptor with nanomolar affinities and block ephrin-induced EphA4 phosphorylation and EphA4-mediated actin remodeling in a growth-cone collapse assay. These results demonstrate the potential of Nbs to selectively target the LBD of the EphA4 receptor. These Nbs may be useful as a therapeutic strategy in disorders in which EphA4 plays a pathogenic role.

## Results

### Generation of anti-EphA4 LBD Nbs

**Figure 1.**
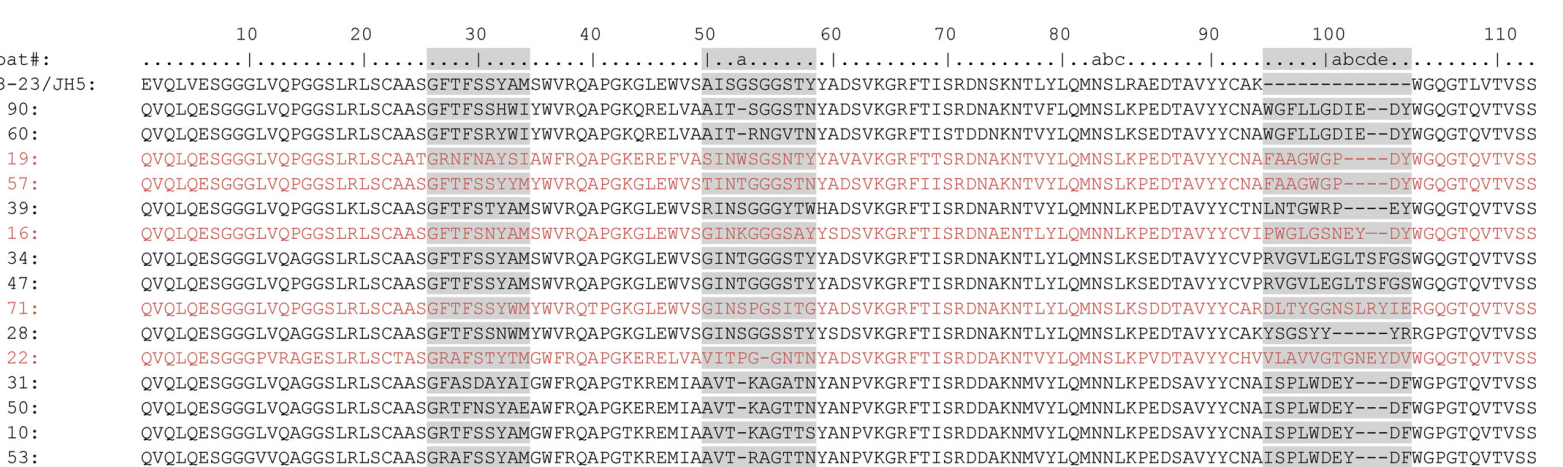
**Amino acid sequence of 15 different anti-EphA4 LBD Nbs.** Nbs were numbered according to Kabat numbering with reference sequence VH3–23/JH5 (human germ line sequence on average most closely related to Nanobody sequences) on *top*. The complementary determining regions were assigned according to the AbM definitions (75) and are shown with a *gray background*. The Nbs belonged to nine clonally unrelated B-cell clones, and the different groups are shown alternatively in *black* and *red*. According to the amino acid sequences Nb 90 and 60, Nb 19 and 57, Nb 34 and 47, and Nb 31, 50, 10, and 53 belonged to the same group. Nb 39, 16, 71, 28, and 22 belonged to unrelated B-cell clones.

An alpaca was immunized with recombinant human EphA4 LBD according to standard procedures (29). In addition to conventional antibodies, alpacas also produce heavy-chain-only antibodies in response to an immunogen. A phagemid library displaying Nbs was constructed from the RNA extracted from peripheral blood lymphocytes and transformed in *Escherichia coli* TG1 cells. A library of ∼2 × 10^8^ independent transformants was obtained, and ∼87% of transformants harbored the vector with the right insert size. Phage particles were generated and subjected to panning. After four consecutive rounds of panning, we obtained 41 colonies that expressed antigen-specific Nbs in their periplasmic extracts, as determined by ELISA. Sequencing of the Nb genes from these 41 positive colonies resulted in 15 different Nbs. Characteristically, Nbs contain three complementary-determining regions that contribute to antigen-binding specificity. Based on sequence homology, they belonged to nine different clonally unrelated B-cell clones (Fig. 1). Nbs belonging to the same group showed very high sequence similarity, suggesting that they are originating from clonally related B-cells that underwent hypermutation. Nbs 39, 16, 71, 28, and 22 most likely belong to unrelated B-cell clones.

### Expression of the anti-EphA4 LBD Nbs

Phagemids encoding the Nbs of interest were transformed into WK6 *E. coli* cells to allow expression of the Nbs without the pIII fusion. The expressed Nbs were recovered from the periplasmic extracts and purified. Their purity was analyzed by Coomassie-stained SDS-polyacrylamide gels, and their identity was determined with Western blot detection using His_6_ tag (data not shown). All Nbs could be detected at a position corresponding to ∼15 kDa. However, there were obvious differences in expression levels of the Nbs (data not shown). Concentration of poorly expressed Nbs 90, 16, 71, 28, and 10 resulted in protein aggregation. Therefore, we did not include these Nbs in further analyses.

### Analysis of EphA4 binding

**Figure 2.**
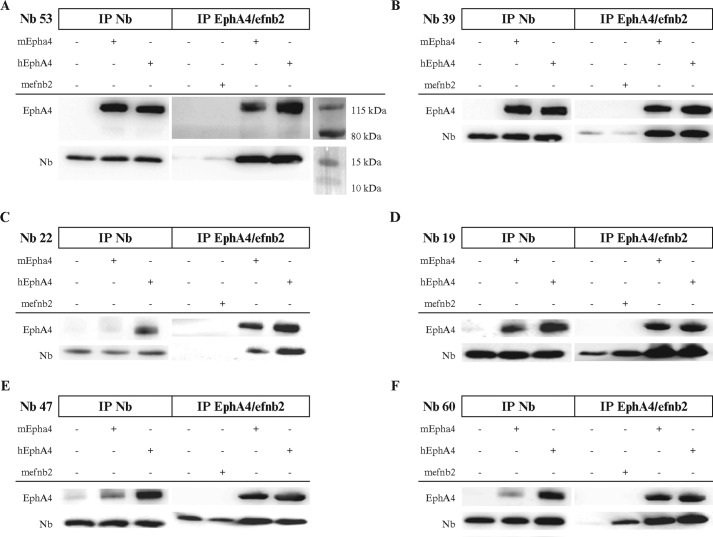
**Nbs bind native EphA4 LBD.** Nbs and EphA4 co-immunoprecipitated (*IP*) together, both when immunoprecipitating different Nbs (*left panel*) or when immunoprecipitating EphA4 (right *panel*). *A* and *B*, Nb 53 and Nb 39 could capture EphA4 and could be captured by EphA4. *C*, Nb 22 showed lower affinity for mouse EphA4 compared with human EphA4. *D* and *E*, Nb 19 and 47 showed unspecific binding to the beads. *F*, Nb 60 showed cross-reactivity with ephrin-B2, which was performed as a negative control experiment.

We next determined whether the Nbs recognized EphA4 under reducing, none-native conditions. None of them bound recombinant EphA4 (data not shown) on Western blot. An EphA4 monoclonal antibody was used as a positive control and readily bound the denatured recombinant protein. These data suggested that the Nbs target a conformational epitope on the EphA4 LBD. To test whether the Nbs were indeed able to bind native EphA4, we performed immunoprecipitation experiments (Fig. 2). Nbs 31, 39, 50, 53, and 57 precipitated EphA4 protein, and EphA4 in turn precipitated these Nbs (data shown only for Nbs 53 and 39 in Fig. 2, *A* and *B*). In contrast to the other Nbs, Nb 22 showed lower binding to mouse EphA4 compared with human EphA4 (Fig. 2*C*). Nbs 19, 34, and 47 revealed nonspecific binding to the beads and to recombinant ephrin-B2 (data shown only for Nb 19 and 47 in Fig. 2, *D* and *E*). Nb 60 did not show nonspecific binding to the beads but precipitated with recombinant ephrin-B2, indicating cross-reactivity with ephrin-B2 (Fig. 2*F*).

To identify the Nbs with the highest affinity, we determined their binding kinetics to EphA4 using surface plasmon resonance (SPR). The EphA4 LBD was immobilized onto the chip and Nbs (1–300 nm) were used as the analyte. SPR analyses revealed that Nbs 34 and 47 do not bind to the EphA4 LBD (Table 1). Nbs 22, 31, 39, 50, 53, 57, and 60 bound in the low nanomolar range, whereas Nb 19 only interacted with EphA4 LBD in the higher nanomolar range (Table 1).

**Table 1:**
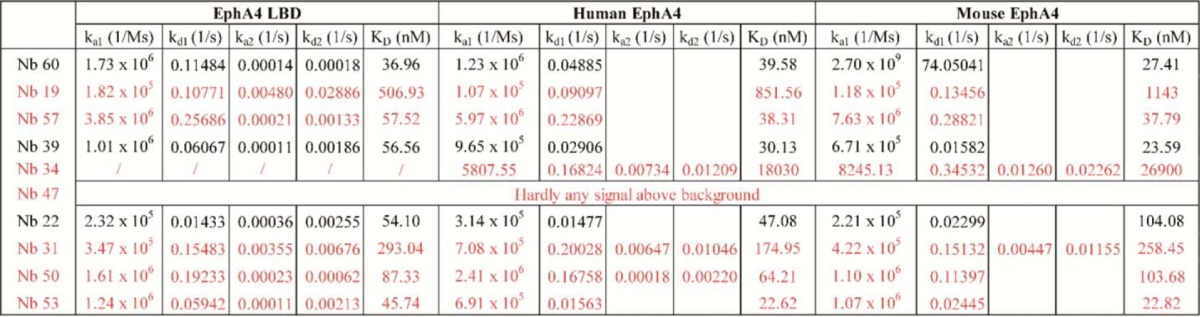
**Overview of the EphA4-Nanobody binding constants** Association constants (k_a1_ and k_a2_), dissociation constants (k_d1_ and k_d2_) and equilibration constants (K_D_) were determined by SPR through kinetics analysis. SPR analyses were performed in duplicate. Alternating red and black colors indicate different Nb groups based on their clonal origin.

We next investigated the affinity of the Nbs for full-length recombinant human and mouse EphA4 receptor. Most Nbs showed similar binding to the full-length receptor as to the EphA4 LBD. Nb 22 showed a 2-fold difference in binding affinity between human and mouse EphA4, in agreement with what we found in immunoprecipitation experiments (Fig. 2*C*). Based on these results, we selected seven Nbs (60, 57, 39, 22, 31, 50, and 53) with affinities in the nanomolar range for both the EphA4 LBD and the full-length receptor.

### Cross-reactivity with other Eph receptors

Because the homology between the different Eph receptors is very high, we used Alphascreen technology to study the specificity of the Nbs generated. All Eph receptors tested showed binding with ephrin-A5 and/or ephrin-B2, indicating that all receptors adopted their correct conformation. Ephrin-A5 interacted with all EphA receptors and with EphB2, as has been previously shown (data not shown) (3). Ephrin-B2 interacted with all EphB receptors and EphA4 but not with EphB6, for which no ligands have been identified so far (data not shown) (4, 30). The control Nb did not bind to any of the Eph receptors as expected (data not shown).

**Figure 3.**
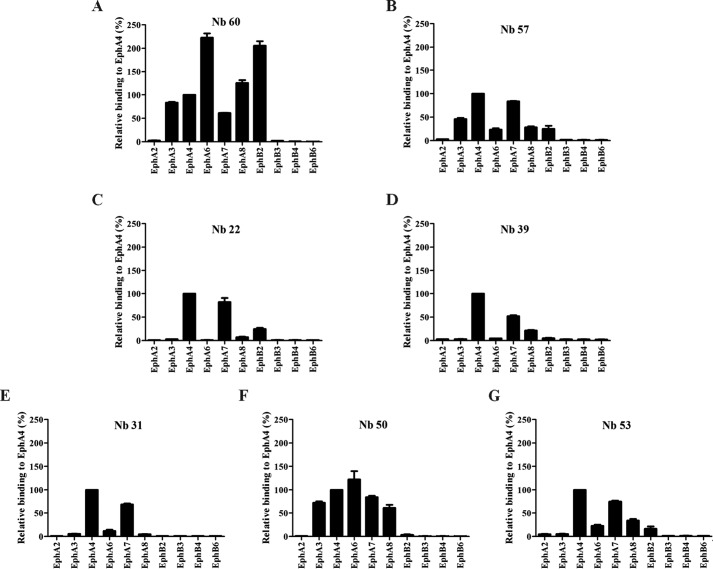
**Selectivity for EphA4 and cross-reactivity with other Eph receptors.** Using AlphaScreen technology we determined the selectivity of the Nbs for EphA4 receptor and the cross-reactivity with other Eph receptors. *A*, next to EphA4, Nb 60 also interacted with many other different Eph receptors. *B–G*, Nb 57, 22, 39, 31, and 53 presented the highest selectivity to the EphA4 receptor. *F*, Nb 50 had the lowest selectivity for EphA4 as it also interacted with EphA3, EphA6, EphA7, and EphA8. All assays were done in duplicate, and data are represented as the mean ± S.D.

Alphascreen analyses showed that Nb 60 interacted with several Eph receptors other than EphA4 (Fig. 3*A*), in agreement with the immunoprecipitation results obtained with this Nb (Fig. 2*F*). Nb 57 bound EphA4 but also EphA3 and EphA7, and Nb 50 bound EphA4 but also EphA3, EphA6, EphA7, and EphA8 (Fig. 3, *B* and *F*). Nbs 22, 31, 39, and 53 were almost completely selective for EphA4, although some binding to EphA7 was detected (Fig. 3, *C*, *D*, *E*, and *G*). We, therefore, selected the latter four for further screening.

### Competition with ephrin ligands for the interaction with EphA4

EphA4 interacts with most of the ephrins (2, 4). We, therefore, investigated whether the selected Nbs were able to displace the ligands from the EphA4 receptor. We determined the optimal concentration for the different ephrin ligands using a fixed concentration of the EphA4 LBD to avoid the hooking effect, and we added increasing concentrations of the Nbs. The control Nb did not compete with any ephrin ligand for the interaction with EphA4, as expected (data not shown). The KYL peptide, a known EphA4 inhibitor, was used as a positive control.

**Figure 4.**
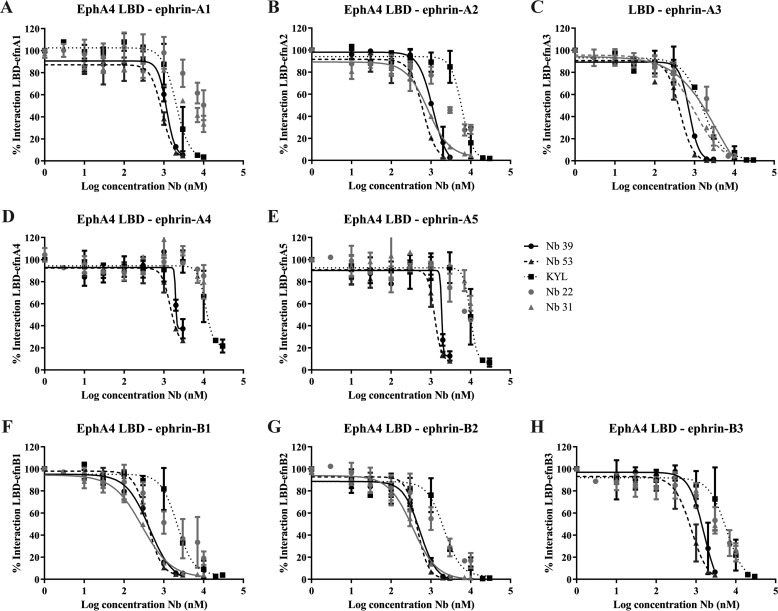
**Nbs compete with ephrin ligands in binding EphA4.** With AlphaScreen technology we determined the capability of the Nbs to inhibit the binding to EphA4 LBD. *A*, *D*, *E*, and *H*, Nb 22 and 31 could not completely inhibit ephrin-ligand binding to EphA4 LBD, whereas Nb 39 and Nb 53 succeeded in doing so even at a lower concentration of the KYL peptide, which was used as a positive control. *B*, *F*, and *G*, Nb 22 did not show complete inhibition, and Nb 39, Nb 53, and Nb 31 showed complete inhibition of ligand binding at a lower concentration of the KYL peptide. *C*, Nb 39 and 53 showed complete inhibition of ligand binding at a lower concentration of Nb 22, Nb 31. and the KYL peptide. Trend-lines are only shown when complete inhibition was reached. All assays were done in duplicate, and data are represented as the mean ± S.D.

Nb 39 and Nb 53 completely displaced all ephrin ligands from EphA4 binding in a concentration range lower than the KYL peptide (Fig. 4, *A–H*). Nbs 22 and 31 were less potent. Nb 22 completely displaced ephrin-A3 (Fig. 4*C*) and almost completely displaced ephrin-B ligands (Fig. 4, *F–H*) from EphA4 binding, but no full displacement of the other ephrin-A ligands was obtained with the concentrations tested (Fig. 4, *A*, *B*, *D*, and *E*). Its potency was comparable to that of the KYL peptide. Nb 31 completely displaced ephrin-A1, ephrin-B1, and ephrin-B2 at lower concentrations than the KYL peptide (Fig. 4, *A*, *F*, and *G*) and completely displaced ephrin-A3 with concentrations similar to the KYL peptide (Fig. 4*C*). However, ephrin-A2, ephrin-A4, ephrin-A5, and ephrin-B3 could not be completely displaced with the concentrations tested (Fig. 4, *B*, *D*, and *E*). These data show that Nbs 39 and 53 were able to block the interaction of all ephrin ligands with the EphA4 LBD.

**Figure 5.**
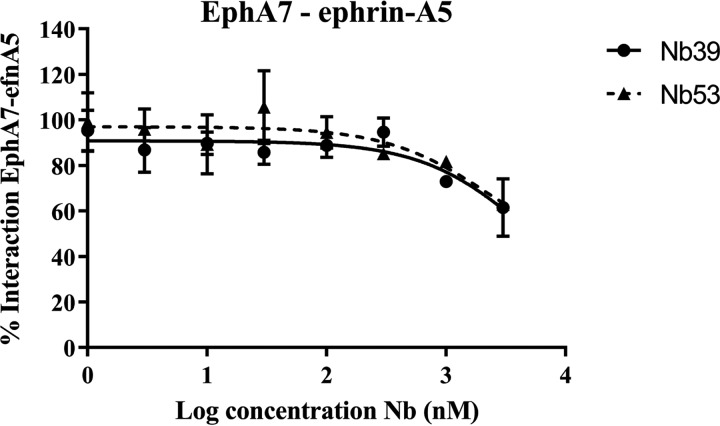
**Nb 39 and Nb 53 do not completely inhibit ephrin-A5 binding to EphA7.** With Alphascreen technology we determined whether Nb 39 and Nb 53 could inhibit ephrin-A5 binding to EphA7. These Nbs did not completely inhibit the interaction between ephrin-A5 and EphA7 at the highest concentrations tested. This assay was done in triplicate, and data are represented as the mean ± S.D.

To examine the potency of Nb 39 and 53 in inhibiting another Eph receptor, EphA7, compared with EphA4, we determined the capacity of these two Nbs to modify the interaction between EphA7 and ephrin-A5 in a competition assay. We determined the optimal binding concentrations of EphA7 and ephrin-A5. At a concentration in which Nb 39 and Nb 53 were able to inhibit binding of ephrin-A5 to EphA4 (Fig. 4*E*), only partial inhibition of the interaction between EphA7 and ephrin-A5 could be detected (Fig. 5). A control Nb did not compete with ephrin-A5-binding to EphA7 (data not shown).

### Inhibition of ephrin-induced EphA4 activation

**Figure 6.**
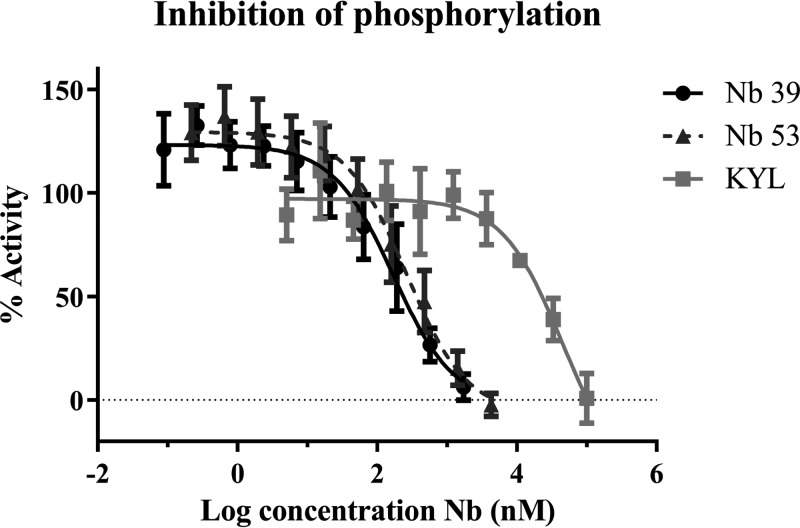
**Nbs inhibit ephrin-induced EphA4 phosphorylation.** With the PathHunter assay we could determine that Nb 39 and Nb 53 completely inhibited EphA4 phosphorylation triggered by ephrin-A1 binding, even at a lower concentration than the KYL peptide. All assays were performed in triplicate, and data are represented as the mean ± S.D.

To assess the antagonistic properties of the Nbs, we tested their effect on ephrin-induced EphA4 phosphorylation (31). Upon ephrin binding, Eph receptors are dimerized and activated by autophosphorylation (phosphorylation of tyrosine residues in the kinase domain and juxtamembrane region) (31). After clustering, the phosphorylated tyrosine residues form binding sites for cytoplasmic targets with Src homology 2 (SH2) or phosphotyrosine binding (PTB) domains (31, 32). In general, EphA4 phosphorylation results in cell-cell segregation, in this way regulating cytoskeleton dynamics and morphology (1). Ephrin-induced phosphorylation was examined using the PathHunter assay in U2OS cells. Cells were stimulated with ephrin-A1 with or without the presence of an EphA4 antagonist, and the resulting EphA4 phosphorylation was measured. The control Nb did not have any effect on receptor phosphorylation (data not shown). The KYL peptide achieved complete inhibition of ephrin-A1-induced phosphorylation with an IC_50_ value of 53 μm (Fig. 6). Nbs 39 and 53 also completely inhibited ephrin-A1-induced phosphorylation at lower concentrations than the KYL peptide, with an IC_50_ of 170 nm and 261 nm, respectively (Fig. 6). These data indicate that Nbs 39 and 53 are 200–300 times more potent than the KYL peptide in inhibiting ephrin-A1-induced phosphorylation of the EphA4 receptor.

### Inhibition of ephrin-induced growth-cone collapse

**Figure 7.**
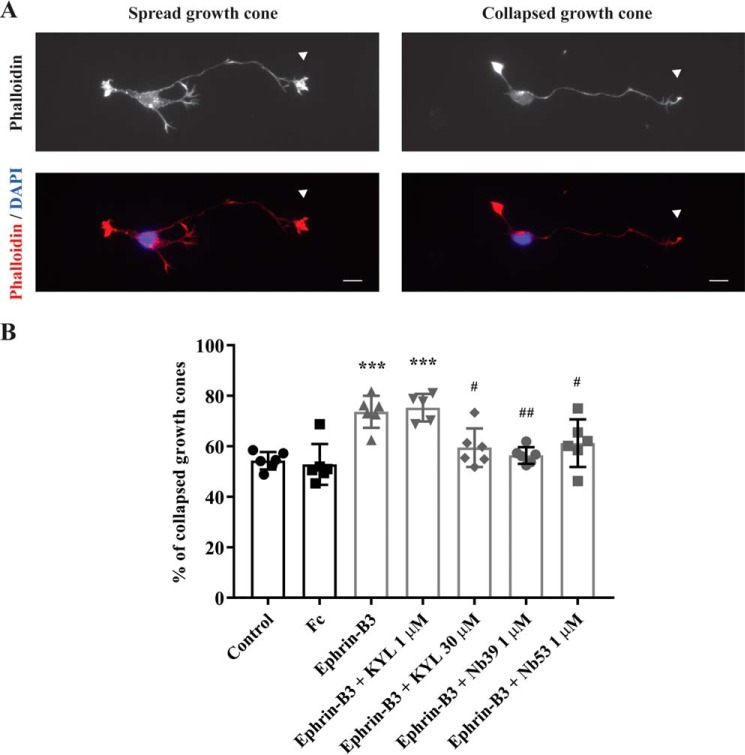
**Nbs inhibit ephrin-B3-EphA4-mediated growth-cone collapse.** Nb 53 and Nb 39 (1 μm) reversed ephrin-B3-mediated growth-cone collapse. *A*, E17.5 cortical neurons were first treated at 24 h after culture with 1 μm or 30 μm KYL peptide or 1 μm Nb for 30 min and then stimulated with 1 μg/ml preclustered ephrin-B3-Fc or Fc as a control for 30 min in the presence of the KYL peptide or the Nb. A control condition without any kind of treatment was also scored (control). Cells were stained with Alexa Fluor 555-conjugated phalloidin and DAPI. Growth-cone collapse was determined for every neuron by analyzing the tip of only the longest neurite (*white arrowheads*). *Scale bar*, 10 μm. *B*, percentage of growth-cone collapse was determined in every experimental condition by assessing 40–160 neurons under a Zeiss fluorescence microscope. Percentage of collapse is expressed as the mean of collapse of six independent experiments ± S.D. and was compared between different conditions by one-way ANOVA followed by Tukey's multiple comparisons post hoc test. ***, *p* < 0.001, as compared with control and Fc conditions. #, *p* < 0.05; ##, *p* < 0.01 as compared with the ephrin-B3 condition.

A common cellular response downstream of Eph receptor activation is actin cytoskeleton reorganization, leading to cell morphology changes (1, 33). Ephrin-B3 treatment of cortical neurons *in vitro* induces collapse of growth cones at the tip of the neurites by reorganization of actin filaments (34, 35). Cortical neurons lacking functional EphA4 show insensitivity to the ephrin-B3-mediated growth-cone collapse, suggesting that this effect is EphA4-mediated (36). We next determined whether the Nbs are also able to antagonize a cellular response such as growth-cone collapse induced by an EphA4 receptor agonist. To this end, we investigated their effect on the ephrin-B3 induced inhibition of neurite elongation in E17.5 embryonic cortical neurons. As described before, stimulation with preclustered recombinant ephrin-B3-Fc resulted in growth-cone collapse as compared with an unstimulated condition and a condition stimulated with Fc only (Fig. 7). As expected, KYL peptide antagonized this effect on growth-cone collapse. Nb 53 as well as Nb 39 also inhibited the growth-cone collapse effect mediated by ephrin-B3-Fc at the concentration of 1 μm, a concentration at which the KYL peptide was ineffective (Fig. 7).

### Nb stability in plasma

**Figure 8.**
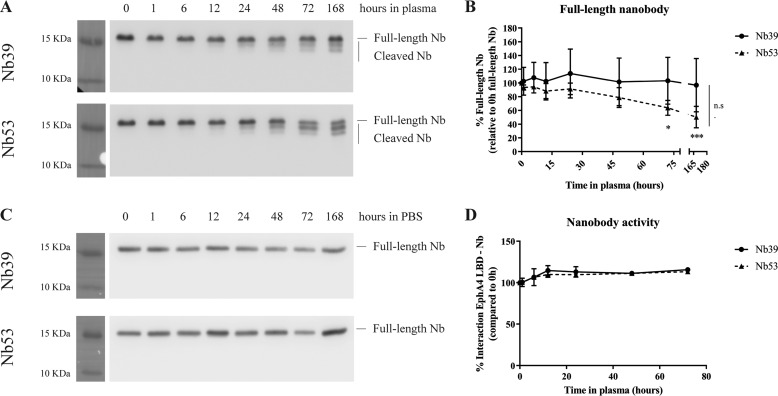
**Nbs are stable in plasma for at least 72 h.** Nbs were incubated in heparinized mouse plasma at 37 °C until a maximum of 168 h. *A*, Western blot against HA tag was performed to detect Nb 39 and Nb 53 full-length and cleavage products. *B*, quantifications of full-length Nb band were normalized to the full-length band at 0h for each Nb. The means of 3–5 independent experiments ± S.D. are represented. Time factor was analyzed by two-way ANOVA followed by Sidak's multiple comparisons post hoc test. *, *p* < 0.05; ***, *p* < 0.001, as compared with the 0-h time point. Differences between the two Nbs were determined by two-way ANOVA but are not identified (*n.s.*). *C*, Nb incubation in PBS did not give rise to degradation products as detected by Western blot. *D*, incubation of Nbs in plasma at 37 °C until a maximum of 72 h did not affect the capability of those in binding EphA4 LBD. All values represent the percentage of Nb-EphA4 LBD binding respect to the 0-h time point. This assay was done in triplicate, and data are represented as the mean ± S.D.

We incubated Nb 39 and Nb 53 in heparinized mouse plasma at 37 °C for up to 168 h (7 days) to study the stability in plasma and obtained aliquots at different time points. We determined the stability of Nbs with Western blot using an anti-HA antibody. Some cleavage products could be observed from 24 h onward for both Nb 39 and 53 (Fig. 8*A*). Whereas full-length Nb 39 remained stable across the time course, full-length Nb 53 levels diminished with time from 72 h onward, suggesting that Nb 53 is more prone to degradation in plasma than Nb 39 (Fig. 8, *A* and *B*). No degradation products were detected for any of the Nbs after incubation in PBS at 37 °C for the same time-course length (Fig. 8*C*). In addition we studied by Alphascreen technology whether the binding capacity of Nbs to EphA4 receptor was altered after incubating the Nbs in plasma. After 72 h of incubation in plasma at 37 °C, Nbs were still capable of binding EphA4 LBD (Fig. 8*D*).

## Discussion

In view of EphA4's role in normal physiology as well as in cancer and neurodegeneration, the generation of potent and selective antagonists against this receptor is of great interest. The EphA4 LBD may be a better target than its tyrosine kinase moiety because of the difficulty to obtain selective Eph tyrosine kinase inhibitors and because of the need for such molecules to enter the cell. Here we show for the first time the generation of single domain antibodies targeting the LBD of EphA4.

Nbs are in many ways superior to small molecules when targeting the LBD of Eph receptor proteins, as high affinity binding of the LBD requires the coverage of the protein interaction surface (900 Å^2^ for EphA4) to block the interaction with EphA4 ligands. Both Nb 39 and Nb 53 were able to block the interaction of EphA4 with all ephrin ligands and to inhibit ephrin-induced phosphorylation of EphA4 and growth-cone collapse triggered by ephrin-B3 binding to endogenous EphA4.

In previous studies Nbs were used to fix receptors in one conformation (28, 37). As a consequence Nbs are an ideal probe to target dynamic structures such as the EphA4 LBD (2, 4, 17). Four different conformations have been reported for the EphA4 LBD: a conformation similar to other EphA receptors bound with ephrin-A ligands (2, 38), a conformation similar to EphB receptors bound with ephrin-B ligands (2, 4, 15), and two unbound conformations when free in solution (17). Fixing the EphA4 LBD in a specific conformation might limit the accessibility for all or a specific class of ephrin ligands, thereby blocking the interaction of those ligands with the LBD.

Nb 39 and Nb 53 were most selective for EphA4 but still showed residual binding to EphA7, suggesting that they target a region that is not highly conserved in all Eph receptors. However, concentrations of Nb 39 and Nb 53 that strongly inhibit EphA4 binding to ephrin ligands only partially reduced the interaction between EphA7 and ephrin-A5. Interestingly, EphA7 KO mice as well as rats treated with EphA7 antisense oligonucleotides showed enhanced recovery after spinal cord injury compared with control mice (39), similar to the effects described for EphA4 antagonists (24). Therefore, if some binding to EphA7 would occur in addition to EphA4, this could potentially be of benefit rather than detrimental.

In the last years extensive work has been performed aiming to develop EphA4 antagonists with high affinity, specificity, and with good pharmacokinetic profiles (14). One EphA4 inhibitor, the KYL peptide, has been studied extensively in models of neurological disorders, such as acute injuries including spinal cord injury and stroke and neurodegenerative disorders such as ALS and Alzheimer's disease (10, 11). Unfortunately, this peptide has a *K_D_* value of ∼1 μm as determined with isothermal titration calorimetry and a very short half-life in serum (11 min in mouse serum) (19, 24). Interestingly, a recent study reported the generation of highly selective EphA4 antagonists with higher potency, the cyclic peptide APY derivatives, APY-d3 and APY-d4 (16, 22). APY-d3 and APY-d4 have *K_D_* values of 30 nm and 20 nm as determined with isothermal titration calorimetry and inhibit ephrin-A5-induced phosphorylation of EphA4 with an IC_50_ of 240 nm and 310 nm. Both cyclic peptides are stable in mouse plasma for >72 h and are currently the best available EphA4 inhibitors (22). Nb 39 and 53 are more potent than the KYL peptide but similarly potent as APY-d3 and APY-d4, with *K_D_* values in the nanomolar range, as measured with SPR. Moreover, both Nbs could inhibit EphA4 phosphorylation with an IC_50_ of 170 nm and 261 nm, respectively. Similar to APY-d3 and APY-d4, Nb 39 and Nb 53 were stable in mouse serum for >72 h and maintained their full capability of binding EphA4 LBD. The affinity of Nbs could still be further increased through error-prone PCR mutagenesis and/or making bispecific Nbs (40, 41). Bivalent or bispecific Nbs can be obtained by connecting two identical or different Nbs with a linker, thereby improving the avidity. However, this strategy requires caution in the setting of developing EphA4 antagonists, as dimerization of Eph receptors may induce clustering and subsequently activation, similar to what is obtained with a preclustered antibody (42).

EphA4 has been found to play a role in cancer biology and in the pathogenesis of several neurological disorders (5). In the central nervous system, EphA4 is highly expressed in cell bodies, dendritic spines, and axons of neurons in various brain regions (43, 44). EphA4 expression is up-regulated in axonal stumps after injury (45) and in sprouting neurons of aged mice after stroke, which contributes to reduced recovery (46). Furthermore, blocking EphA4 induced more sprouting after spinal cord injury (8). EphA4's negative effect on axonal regeneration is mediated through the interaction of the receptor with ephrin ligands on surrounding cells such as muscle cells, astrocytes, microglia, and oligodendrocytes (47). Neuronal EphA4 may interact with ephrin-A5 and ephrin-B2, of which the expression is highly up-regulated in reactive astrocytes after injury (48–50). This high astrocytic ephrin-A5 expression inhibited axonal sprouting and motor recovery after stroke (49). Also, ephrin-B2 expression decreased axonal sprouting, as deleting ephrin-B2 from reactive astrocytes reduced glial scar formation and improved recovery after spinal cord injury, and this was correlated with an increased regenerative capacity of sprouting spinal cord axons (45, 48, 50). Oligodendrocytes precursor cells are glial cells responsible for remyelination during neuronal injury or degeneration, thereby inhibiting axonal outgrowth (51). Neuronal EphA4 may interact with ephrin-A1, ephrin-A3, ephrin-A5, ephrin-B1, and ephrin-B2, which have been identified in oligodendrocyte precursor cells and in mature oligodendrocytes (52–54). In addition, enhanced functional recovery was observed in ephrin-B3 KO mice after spinal cord injury, and blocking ephrin-B3 promotes remyelination *in vivo* in a rat model of remyelination (55, 56). The relative importance of the different surrounding cells and different ligands is not yet known. However, as EphA4 is a promiscuous receptor, targeting EphA4 by blocking the interaction with all ephrin ligands might be the most efficient therapeutic strategy against neurological diseases.

Treating neurological disorders by targeting EphA4 implies using substances that are able to penetrate the blood brain barrier (BBB) to reach their target. In acute injuries, this barrier is impaired. In neurodegenerative diseases such as ALS, getting drugs across the BBB remains a major challenge despite the fact that the BBB is abnormal in this disease (57–59). Several strategies to get biologics across the BBB have been developed, such as transcytosis through clathrin vesicles and receptor-mediated transcytosis by targeting the low-density lipoprotein receptor, the transferrin receptor, or the insulin receptor (60–65). Interestingly, one study showed that Nbs with a high isoelectric point can cross the BBB spontaneously (66).

Nbs also have some drawbacks. Although Nb 39 and Nb 53 remained stable in mouse plasma for >72 h, the small size of Nbs limits their half-life to ∼1.5 h after *in vivo* administration (67). Due to their small size they are rapidly cleared from blood via the kidney (27). This low half-life can be overcome by linking the Nb to serum albumin, which can increase the half-life to 20–30 h in mice (68). In humans, this approach extended the half-life of Nbs to 19 days (68, 69). Another strategy to increase the half-life of Nbs is coupling polyethylene glycol (PEG) groups to the Nbs (70). The addition of PEG groups increases the apparent molecular weight above the glomerular filtration limit, avoiding renal clearance and/or evades cellular clearance mechanisms (71).

In summary, we describe for the first time the development of Nbs that target the EphA4 LBD. We identified two Nbs that were specific for EphA4 with residual EphA7 binding and with *K_D_* and IC_50_ values in the nanomolar range. Both Nbs were able to block the interaction of EphA4 with all ephrin ligands and inhibit EphA4 phosphorylation and the growth-cone collapse mediated by EphA4 activation upon ephrin-B3 interaction. These Nbs may be useful tools to study the role of the EphA4 receptor in preclinical cancer or neurodegeneration models. Furthermore, they may represent a starting point for an EphA4-based therapeutic approach using Nbs.

## Experimental procedures

### Expression and purification of the EphA4 ligand-binding domain

The EphA4 LBD (amino acids 22–203; Ref. 17) was cloned from the EphA4 Human cDNA ORF clone (Origene) and expressed in the *E. coli* strain BL21 codon + pICA2 transformed with the pLH36Epha plasmid. Expression was induced by isopropyl β-d-1-thiogalactopyranoside under control of a pL-promotor developed by the Protein Service Facility of VIB. The pLH36 plasmid was provided with a His_6_ tag followed by a murine caspase-3 site. The murine caspase-3 site can be used to remove the His_6_ tag attached at the N terminus of the protein of interest during purification. The transformed bacteria were grown in Luria Bertani medium supplemented with ampicillin (100 μg/ml) and kanamycin (50 μg/ml) overnight at 28 °C inoculation (1/100) in a 20-liter fermenter provided with Luria Bertani medium supplemented with ampicillin (100 μg/ml) and 1% glycerol. The initial stirring and airflow was 200 rpm and 1.5 liters/min, respectively. Furthermore, this was automatically adapted to keep the *p*O_2_ at 30%. The temperature was kept at 28 °C. The cells were grown to an optical density of *A*_600 nm_ = 1.0 and transferred at 20 °C, and expression was induced by the addition of 1 mm isopropyl β-d-1-thiogalactopyranoside overnight. Cells were then harvested and frozen at −20 °C. After thawing, the cells were resuspended at 3 ml/g in 20 mm NaH_2_PO_4_, pH 7.5, 500 mm NaCl, 20 mm imidazole, and 1 mm phenylmethylsulfonyl fluoride. The cytoplasmic fraction was prepared by sonication of the cells followed by centrifugation at 18,000 × *g* for 30 min. All steps were conducted at 4 °C. The clear supernatant was applied to a 74-ml Ni^2+^-Sepharose 6 FF column (GE Healthcare), equilibrated with 20 mm NaH_2_PO_4_, pH 7.5, 500 mm NaCl, 20 mm imidazole, and 0.1% CHAPS. The column was eluted with 20 mm NaH_2_PO_4_, pH 7.4, 20 mm NaCl, 400 mm imidazole, and 0.1% CHAPS after an extra wash step with 50 mm imidazole. The elution fraction was diluted 120 with 20 mm Tris, pH 8.0, and 0.1% CHAPS and loaded on a 20-ml Source 15Q column (GE Healthcare) to remove contaminants. After equilibration, the protein of interest was eluted by a linear gradient over 20 column volumes of NaCl from 0 to 1 m in 20 mm Tris, pH, 8.0 and 0.1% CHAPS. To the EphA4-containing fractions, activated murine caspase-3 (1/100% murine caspase-3/Epha4) with 10 mm DTT was added to remove the His_6_ tag. After a 1-h incubation at 37 °C, the reaction solution was injected on a HiLoad 26/60 Superdex 75 prep grade with PBS as the running solution for formulation and removal of minor contaminants, His_6_ tag and murine caspase-3. The obtained fractions were analyzed by SDS-PAGE, and the concentration was determined using the Micro-BCA assay (Thermo Fisher Scientific, Ghent, Belgium).

### Construction of a VHH library

Nbs targeting the EphA4 LBD were obtained in collaboration with the VIB Nanobody Service Facility. An alpaca was injected subcutaneously on days 0, 7, 14, 21, 28, and 35, each time with 250 μg of human EphA4 LBD. On day 39 anticoagulated blood was collected for lymphocyte preparation. A VHH library was constructed as previously described (72–74) and screened for the presence of antigen-specific Nbs. In brief, total RNA from peripheral blood lymphocytes was used as the template for first-strand cDNA synthesis with the oligo(dT) primer. Using this cDNA, the VHH-encoding sequences were amplified by PCR, digested with PstI and NotI, and cloned into the PstI and NotI sites of the phagemid vector pMECS. A VHH library of ∼2 × 10^8^ independent transformants was obtained.

### Isolation of hEphA4 LBD-specific Nbs

To screen for the presence of EphA4-specific Nbs, the library was subjected to four consecutive rounds of panning, performed on solid-phase-coated EphA4 LBD (concentration: ∼200 μg/ml, ∼20 μg/well). The enrichment for antigen-specific phages after each round of panning was assessed by comparing the number of phages eluted from antigen-coated wells with the number of phages eluted from negative control (only-blocked) wells. These experiments suggested that the phage population was enriched for antigen-specific phages after the 3rd and 4th round of panning. In total, 190 individual colonies from the 3rd and 4th round (95 from each round) were randomly selected and analyzed by ELISA for the presence of antigen-specific Nbs in their periplasmic extracts (ELISA using crude periplasmic extracts including soluble Nbs). Of 190 colonies, 41 colonies (14 and 27 from the 3rd and 4th round, respectively) scored positive in this assay. The VHH genes of the selected genes were sequenced to identify the different Nbs.

### Expression and purification of recombinant Nbs

pMECS vectors harboring Nb genes were transformed into WK6 *E. coli* cells, and the transformed cells were grown in Luria Bertani medium supplemented with ampicillin (100 μg/ml) and 0.1% glucose at 37 °C overnight. Subsequently, the cultures were inoculated 1/100 to have 1-liter productions in Terrific Broth medium supplemented with ampicillin (100 μg/ml) and 0.1% glucose in baffles shake flasks. The temperature was kept at 37 °C. The cells were grown to an optical density of *A*_600 nm_ = 1.0 and transferred at 28 °C, and expression was induced by the addition of 1 mm isopropyl β-d-1-thiogalactopyranoside overnight. Cells were then harvested and frozen at −20 °C. The expressed Nbs were extracted from the periplasm by osmotic shock and purified using His GraviTrap (GE Healthcare) in parallel, equilibrated with 20 mm NaH_2_PO_4_, pH 7.5, 300 mm NaCl, 20 mm imidazole, and 1 mm PMSF. After loading, the columns were washed with 20 column volumes of the same buffer. The Nbs were first eluted with 20 mm NaH_2_PO_4_, pH 7.5, 20 mm NaCl, 50 mm imidazole, 1 mm PMSF and then with 400 mm imidazole in the same buffer. Finally, the Nbs were desalted to PBS on Sephadex G25 (GE Healthcare). The obtained fractions were analyzed with Coomassie-stained SDS-polyacrylamide gels. Protein concentration was measured by the Micro-BCA assay (Thermo Fisher Scientific).

### Western blot

For immunoprecipitation experiments, 1 μg of EphA4 LBD, hEphA4-Fc (R&D Systems, Abingdon, UK), and mEphA4-Fc (R&D Systems) were boiled for 10 min in reducing sample buffer (Thermo Fisher Scientific), and proteins were separated in a 4–12% Bis-Tris SDS-PAGE gel (Thermo Fisher Scientific). After SDS-PAGE, the gel was transferred to Immobilon-P membrane (Merck Millipore, Overijse, Belgium) and subsequently blocked with 10% Blotting-grade blocker (Bio-Rad) for 1 h at room temperature. One μg Nb and mouse anti-HA antibody 1/1000 (clone 16B12, MMS-101P-200, lot D13FF01646, Covance) were used to detect EphA4. To determine Nb stability, 10 ng of Nbs were denatured with reducing sample buffer (Thermo Fisher Scientific) and separated in a 12% Tris-glycine SDS-PAGE gel. The gel was transferred to Immobilon-P membrane, which was afterward blocked as described above. To detect the Nbs, 1/1000 anti-HA antibody (clone C29F4, 3724S, lot 8, Cell Signaling) was used.

### Immunoprecipitation experiments

1.5 mg of protein G magnetic Dynabeads (Thermo Fisher Scientific) was preblocked with 1% BSA for 1 h at room temperature, washed 4 times with PBS, and incubated with 2.5 μg of recombinant mouse or human EphA4 protein (R&D Systems) for 10 min at room temperature. After washing 4 times with PBS the beads were incubated with 1 μg of Nb overnight at 4 °C. The beads were washed 4 times with PBS and boiled for 10 min in reducing sample buffer (Thermo Fisher Scientific), and proteins were separated in a 4–12% Bis-Tris SDS-PAGE gel (Thermo Fisher Scientific). After SDS-PAGE, the gel was transferred to Immobilon-P membrane (Merck Millipore) and subsequently blocked with 10% Blotting-grade blocker (Bio-Rad) for 1 h at room temperature. Mouse N-terminal anti-EphA4 antibody 1/1000 (EM2801, lot 1, ECM Biosciences) and mouse anti-HA antibody 1/1000 (clone 16B12, MMS-101P-200, lot D13FF01646, Covance) were used to detect EphA4 and Nb, respectively. To capture EphA4 with Nb anti-HA magnetic Dynabeads (Thermo Fisher Scientific) were used, and all the following steps were performed as described above.

### Stability experiments

To determine the Nb stability in plasma, Nbs were incubated at 37 °C at a concentration of 10 ng/μl in sodium heparin not-filtered C57BL/6 mouse plasma (BioreclamationIVT). At every time point an aliquot was collected and diluted 110 in PBS for further Western blot analysis. As a control, Nbs were incubated for the same time and at the same concentration at 37 °C in PBS. Nb stability in all samples was next determined with Western blot as described above. Statistical analysis was performed using the two-way ANOVA followed by Sidak's multiple comparisons post hoc test as indicated in the figure legends. A 95% confidence interval was used, and values of *p* < 0.05 were considered as statistically significant.

To determine functional stability of Nbs, they were first incubated at 37 °C in sodium heparin not-filtered C57BL/6 mouse plasma at 500 nm concentration. At every time point and for a maximum of 72 h, an aliquot was collected and stored for further analysis with Alphascreen technology.

### Surface plasmon resonance

The equilibrium dissociation constant (*K_D_*) and the association (*k_a_*_1_ and *k_a_*_2_) and dissociation rates (*k_d_* and *k_d_*_2_) were determined using surface plasmon resonance detection on a BIACore T200 (GE Healthcare). Two approaches were used. First, the extracellular N-terminal domain of human EphA4 was immobilized directly onto a CM5 S series sensor chip (GE Healthcare) using standard amine coupling. After activation of the carboxyl moieties on the matrix on the chip surface with a 7-min injection of a 1:1 mixture of 0.4 m EDC (l-ethyl-3-(3-dimethylaminopropyl)carbodiimide) and 0.1 m NHS, the N-terminal domain of human EphA4 (50 μg/ml in 10 mm acetate, pH 4.5) was immobilized to a predefined level of 300 response units (RU). A flow channel activated with EDC (l-ethyl-3-(3-dimethylaminopropyl)carbodiimide)/NHS and immediately afterward blocked by ethanolamine served as reference channel. Throughout the analysis 10 mm Hepes, 150 mm NaCl, 3 mm EDTA, and 0.01 mm Surfactant P-20, pH 7.35, was used as the running buffer. 20 mm glycine, pH 2.5, was used to regenerate the channels (remove all bound proteins). The second approach used the anti-human Fc capture kit as described by the manufacturer. Briefly, ∼8000 RU anti-human Fc antibody was captured on the surface of the channels using amine coupling as described above. In a next step, EphA4-hFc (both human and mouse EphA4) (R&D Systems) fusion protein (2 μg/ml diluted in running buffer) was injected (10 μl/min for 6 min) over the channel resulting in the capture of ∼500 RU of EphA4-hFc fusion protein on the surface of the channel. In this approach, a channel with only anti-human Fc antibody served as the reference. 3 m MgCl_2_ was used as regeneration buffer. The Nbs were diluted to the indicated concentrations in running buffer and injected (60 μl/min) over the channel with immobilized EphA4 and the reference channel. After correction of the response using the responses from the reference channel and a blank injection of running buffer over the Eph4A-immobilized channel (double referencing), kinetic parameters were determined using Biacore T200 evaluation software (GE Healthcare). Interactions of Nbs with different EphA4 recombinant proteins were calculated with a 1:1 binding model or a two-state model. The latter model was used to calculate all Nb interactions with EphA4 LBD and interactions of Nb 34, 31, and 50 with human and mouse EphA4.

### AlphaScreen

To test the specificity of the Nbs for the different Eph receptors, all Nbs were biotinylated with a five times molar excess of EZ link NHS biotin (Thermo Fisher Scientific). The Nbs were incubated with the biotin for 2 h on ice allowing the interaction of the biotin with the primary amines on the surface of the protein. To remove unbound biotin, dialysis was performed with PBS in the Slide-A-Lyzer mini dialysis device (10-kDa cutoff, Thermo Fisher Scientific). Ten microliters of biotinylated Nbs (100 nm) were incubated with 10 μl of a subhooking concentration of mouse recombinant Eph receptor (Fc-tagged; R&D Systems) for 1 h in standard buffer (50 mm Hepes, 100 mm NaCl, 0.1% Triton, and 0.1% BSA) in white opaque 384-well microplates (PerkinElmer Life Sciences) to avoid the hooking effect (oversaturation of donor and acceptor beads inhibiting their association). The determined subhooking concentrations were 10 nm for EphA2, A3, A4, A6, B2, and B6, 30 nm for EphA7 and B3, B4, and 100 nm for EphA8. Subsequently we incubated first 1 h with 10 μl of anti-IgG AlphaLISA acceptor beads (20 μg/ml, PerkinElmer Life Sciences) and then incubated an additional 30 min with 10 μl of streptavidin donor beads (20 μg/ml, PerkinElmer Life Sciences). Incubation steps were performed at room temperature and protected from light.

To test the inhibition of interaction between EphA4 and its different ligands, the EphA4 LBD (2 mg/ml) was biotinylated with a 2.5× molar excess of EZ link NHS biotin (Thermo Fisher Scientific) for 2 h on ice, allowing the interaction of the biotin with primary amines on the surface of the protein. To remove unbound biotin, dialysis was performed with PBS in the Slide-A-Lyzer mini dialysis device (10-kDa cutoff, Thermo Fisher Scientific). Five microliters of a subhooking concentration of biotinylated EphA4 LBD (10 nm) was incubated with 5 μl of different concentrations of Nb for 1 h in standard buffer (50 mm Hepes, 100 mm NaCl, 0.1% Triton, and 0.1% BSA). Subsequently, 5 μl of a subhooking concentration of recombinant ephrin ligand (Fc-tagged; R&D Systems) was added and incubated for 1 h at room temperature. The determined subhooking concentrations were 3 nm for ephrin-A1 and ephrin-A4 and 10 nm for ephrin-A2/3, ephrin-A5, and ephrin-B1–3. Next, 5 μl of anti-IgG AlphaLISA acceptor beads (20 μg/ml; PerkinElmer Life Sciences) and 5 μl of streptavidin donor beads (10 μg/ml; PerkinElmer Life Sciences) were added and incubated for 1 h and 30 min, respectively, at room temperature, protected from light. A Nb targeting superoxide dismutase 1 (SOD1) was used as negative control in all Eph-binding assays.

To test the inhibition of EphA7 and ephrin-A5 interaction, His-tagged human ephrin-A5 (Sino Biological, Beijing, China) was biotinylated with a five-times molar excess of EZ link NHS biotin (Thermo Fisher Scientific) as is described for the Nbs. Briefly, ephrin-A5 was incubated with the biotin for 2 h on ice and dialyzed in PBS with the Slide-A-Lyzer mini dialysis device (10-kDa cutoff, Thermo Fisher Scientific) to remove unbound biotin. Five microliters of a subhooking concentration of human EphA7 (3 nm) was incubated with 5 μl of different concentrations of Nb for 1 h in standard buffer (50 mm Hepes, 100 mm NaCl, 0.1% Triton, and 0.1% BSA). Next, 5 μl of subhooking concentration of biotinylated ephrin-A5 ligand (30 nm) was added and incubated for 1 h at room temperature. Finally, 5 μl of anti-IgG AlphaLISA acceptor beads (20 μg/ml; PerkinElmer Life Sciences) and 5 μl of streptavidin donor beads (20 μg/ml; PerkinElmer Life Sciences) were added and incubated for 1 h and 30 min, respectively, at room temperature, protected from light. A control Nb targeting chicken lysozyme was used as negative control, and untagged ephrin-A5 was used as positive control.

To determine the capability of Nbs to bind EphA4 after plasma incubation, 500 nm Nbs preincubated in plasma were first diluted in PBS at the time of the Alphascreen assay to reach a final subhooking Nb concentration of 10 nm. Five microliters of a subhooking concentration of biotinylated EphA4 LBD (10 nm) was incubated with 5 μl of 10 nm Nb 39 and Nb 53 for 1 h in standard buffer (50 mm Hepes, 100 mm NaCl, 0.1% Triton, and 0.1% BSA). Next, 5 μl of anti-HA AlphaLISA acceptor beads (20 μg/ml; PerkinElmer Life Sciences) and 5 μl of streptavidin donor beads (10 μg/ml; PerkinElmer Life Sciences) were added and incubated for 1 h and 30 min, respectively, at room temperature, protected from light. As a control, to assess the specificity of the binding, 300 nm ephrin-B2 ligand (Fc-tagged; R&D Systems) was incubated with EphA4 LBD for 1 h before the addition of the Nbs.

Plates were read on the Envision Multilabel Reader (PerkinElmer Life Sciences).

### Phosphorylation assay

The amount of phosphorylation of EphA4 was determined using the PathHunter assay (DiscoveRx Corp., Birmingham, UK) with U2OS cells adapted for the EphA4 receptor according to manufacturer's instructions. In short, a small peptide epitope is expressed recombinantly on the intracellular C terminus of the EphA4 receptor-tyrosine kinase. An interaction partner containing SH2 domains is co-expressed with a larger sequence, termed enzyme activator (EA). Activation with recombinant human ephrin-A1-Fc causes EphA4 receptor dimerization, leading to cross-phosphorylation of tyrosine residues on the cytoplasmic domain of the receptor. The SH2-EA fusion protein binds the phosphorylated receptor enabling the complementation of EA and the peptide epitope, yielding an active β-galactosidase enzyme. This interaction can be visualized with a chemiluminescent substrate. Increasing concentrations of the Nbs were added to the medium before ephrin-A1-Fc stimulation. 20 μl of 5000 EphA4-expressing U2OS cells were plated in 384-well plates and incubated for 24 h at 37 °C 5% CO_2_. Five-μl Nb dilutions or vehicle were added per well followed by incubation for 1 h at 37 °C. Five μl of ephrin-A1-Fc (1.2 μg/ml) or vehicle were added to each well followed by incubation for 3 h at room temperature. Twelve μl of detection reagent (Galacton Star, Emerald II solution, PathHunter cell assay buffer in relative volumes of 1:5:19, respectively) was added, incubated for 1 h at room temperature, and read on a Pherastar (BMG Labtech, Ortenberg, Germany). Percentage activity was calculated as (signal − non-stimulated control (no ephrin added))/(ephrin-stimulated condition − non-stimulated control)) × 100. Positive and negative controls were Dasatinib and a Nb targeting Superoxide Dismutase 1 (SOD1), respectively.

### Growth-cone collapse

The cortex of E17.5 mouse embryos was dissociated by trypsinization and trituration. Cortical neurons were cultured on 13-mm-diameter coverslips coated with poly-l-lysine, at a density of 15,000 cells/coverslip in minimum Eagle's medium (MEM) with Earle's salt and l-glutamine and supplemented with 10% heat-inactivated horse serum and penicillin and streptomycin (Thermo Fisher Scientific). Cultures were kept in a 5% CO_2_ humidified incubator at 37 °C. Medium was replaced for Neurobasal medium supplemented with B27, 500 μm l-glutamine and penicillin and streptomycin (Thermo Fisher Scientific) 4 h after plating. 24 h after plating the cultures were incubated for 30 min with KYL peptide at a concentration of 1 μm or 30 μm or Nb 53 or Nb 39 at a concentration of 1 μm. Cultures were next stimulated with 1 μg/ml mouse preclustered ephrin-B3-Fc, or Fc (R&D Systems) as a control and for 30 min in the presence of the KYL peptide or the Nbs. Cortical neurons were fixed for 20 min in 4% paraformaldehyde (Thermo Fisher Scientific), permeabilized in 0.2% Triton X-100 in PBS, and stained with Alexa Fluor 555-conjugated phalloidin (Thermo Fisher Scientific). Growth-cone collapse was scored for each condition under a Zeiss Axioimager M1 epifluorescence and brightfield upright microscope with a Zeiss A-Plan 40×/0.65 ∞/0.17 objective (Carl Zeiss, Oberkochen, Germany). Growth-cone collapse was considered when no lamellipodia or filopodia were present at the tip of the longest neurite of every scored neuron. Growth-cone collapse was scored in a blinded manner for 40–160 neurons in every condition and experiment. Images were obtained with Axiocam MRm monochrome digital camera and Zeiss AxioVision V 4.8.2.0 software (Carl Zeiss) at the same magnification. Statistical analysis was performed using one-way ANOVA followed by Tukey's post hoc test as indicated in the figure legends. A 95% confidence interval was used, and values of *p* < 0.05 were considered as statistically significant.

## Author contributions

L. S. and L. R. designed, performed, and analyzed most of the experiments and wrote the manuscript. B. R., M. T., S. L., A. C., P. J., and P. W. designed, performed, and analyzed some experiments. L. C. G. and M. D. assisted with the analysis of results. J. H. expressed, purified, and isolated the N-terminal EpA4 protein and the Nbs. G. H. G. generated the anti-EphA4 LBD Nbs. B. D. S., P. V. D., L. V. D. B., and R. L. supervised the experiments and contributed to the analysis. W. R. conceived the idea for the project, supervised the experiments, and wrote the manuscript. All authors contributed to the final manuscript.

## Footnotes

9 The abbreviations used are:

ALS: amyotrophic lateral sclerosis
LBD: ligand-binding domain
Nb(s): Nanobody(ies)
SPR: surface plasmon resonance
SH2: Src Homology 2
BBB: blood brain barrier
Bis-Tris: 2-[bis(2-hydroxyethyl)amino]-2-(hydroxymethyl)propane-1,3-diol
ANOVA: analysis of variance
RU: response units
EA: enzyme activator
E: embryonic day.
